# High-dose radiation induces dendritic cells maturation by promoting immunogenic cell death in nasopharyngeal carcinoma

**DOI:** 10.3389/fimmu.2025.1554018

**Published:** 2025-02-18

**Authors:** Xianlin Zeng, Xianhuai Jin, Ji Leng, Shuai Zhang, Yun Wang, Jin Chen, Shichao Zhang, Lijing Teng, Zuquan Hu, Shi Zhou, Zhu Zeng, Jinhua Long

**Affiliations:** ^1^ School of Biology and Engineering/School of Basic Medical Sciences, Guizhou Medical University, Guiyang, China; ^2^ Engineering Center of Cellular Immunotherapy of Guizhou Province, Guiyang, China; ^3^ Key Laboratory of Infectious Immunity and Antibody Engineering of Guizhou Province, Guiyang, China; ^4^ Department of Oncology, Guiyang Public Health Clinical Center, Guiyang, Guizhou, China; ^5^ Department of Oncology, School of Clinical Medicine, Guizhou Medical University, Guiyang, Guizhou, China; ^6^ Department of Interventional Radiology, Affiliated Hospital of Guizhou Medical University, Guiyang, China; ^7^ Key Laboratory of Endemic and Ethnic Diseases, Ministry of Education, Guizhou Medical University, Guiyang, China; ^8^ State Key Laboratory of Functions and Applications of Medicinal Plants, Guizhou Medical University, Guiyang, China; ^9^ Department of Oncology, Affiliated Hospital of Guizhou Medical University, Guiyang, Guizhou, China; ^10^ Department of Oncology, Affiliated Cancer Hospital of Guizhou Medical University, Guiyang, Guizhou, China

**Keywords:** radiotherapy, nasopharyngeal carcinoma, immune response, immunogenic cell death, dendritic cells

## Abstract

**Aim and background:**

Due to the radiosensitivity and deep anatomical location of nasopharyngeal carcinoma (NPC), radiotherapy serves as the cornerstone of standardized treatment for this malignancy. Beyond its cytotoxic effects, radiotherapy can serve as an immunological adjuvant by inducing immunogenic cell death (ICD). Dendritic cells (DCs), as potent antigen-presenting cells, play a critical role in tumor immunotherapy, but their exact role in the ICD process of NPC remains unclear. The effects of high-dose radiation (≥2 Gy) on DCs and the type of immune response it elicits in NPC have not been fully elucidated.

**Methods:**

An *in vitro* study was conducted to assess whether ICD of NPC 5-8F cells induced by high-dose radiation could regulate the immune response of DCs. Specifically, the maturation and antigen-presenting capacity of DCs were evaluated following co-culture with NPC cells exposed to high-dose radiation.

**Results:**

High-dose radiation was found to induce ICD in NPC 5-8F cells, as evidenced by increased pro-inflammatory factor levels and reduced anti-inflammatory factor levels in the cell culture supernatant. Co-culture with NPC cells exposed to high-dose radiation for 15 minutes significantly enhanced the expression of surface molecules on DCs, promoting their immune sensitization.

**Conclusion:**

High-dose radiation-induced apoptosis of NPC 5-8F cells is a form of ICD, which plays an important role in regulating DC immune function. These findings provide insight into the immunomodulatory effects of radiotherapy in NPC and its potential to enhance tumor immunotherapy through DC activation.

## 1 Introduction

Nasopharyngeal carcinoma (NPC) is an epithelial carcinoma originating from the nasopharyngeal mucosa, commonly found in Southeast Asia, East Africa, and North Africa (1). Due to the complex anatomical structure of the nasopharynx and its proximity to important blood vessels and nerves, early-stage NPC is typically treated with radiotherapy alone, while local advanced cases undergo comprehensive treatment with radiotherapy as the main approach (2). For recurrent or metastatic NPC, chemotherapy is the primary treatment modality (3). With continuous advancements in radiotherapy techniques and pharmacological treatments, the five-year survival rate for patients with NPC can reach approximately 70-80% (4). However, some patients experience local treatment failure and/or distant metastasis due to the development of radiotherapy resistances (5), which represents a significant bottleneck in the current treatment of NPC.

Radiotherapy is a therapy that uses ionizing radiation (IR) to damage the DNA structure of tumor cells, thereby inhibiting their proliferation (6). Previously, radiotherapy was believed to only exert immunosuppressive effects on the body, but more recent studies have revealed that radiotherapy can also promote antitumor immune responses, thereby acting as an immunoadjuvant (7, 8). Current research indicates that radiotherapy primarily exerts its immunoadjuvant effects through three mechanisms: Firstly, radiotherapy can induce the release of tumor cell DNA, activating the STING pathway, which promotes the expression of type I interferon, leading to the activation of CD8^+^ T cells and a cytotoxic T lymphocyte antitumor response (6, 9). Secondly, radiotherapy can enhance T cell recognition and cytotoxicity by inducing the release of interferons and chemokines (10, 11). Thirdly, radiotherapy can induce immunogenic cell death (ICD) in tumor cells, which is a regulated form of cell death, thereby promoting DC maturation and infiltration into tumor tissues, ultimately activating specific antitumor immune responses (12, 13). This process enhances DC antigen presentation by releasing damage-associated molecular patterns (DAMPs) from cancer cells, which ultimately activate adaptive responses of CD8^+^ T cells (12, 14). DAMPs include molecules such as high-mobility group box 1 (HMGB1), calreticulin (CRT), heat shock proteins 70 (HSP70) (15), among which HSP70 can be recognized by DCs through CD91, promoting DC maturation and the secretion of pro-inflammatory cytokines like TNF-α (14); CRT acts as a phagocytic signal for DCs, facilitating DC recruitment and antigen uptake; HMGB1 is a histone-chromatin binding protein released from the nuclei of tumor cells after radiation damage. It can bind to TLR4, upregulating the expression of surface molecules on DCs (such as CD80, CD83, and CD86), thereby promoting DC maturation and the proliferation and activation of T cells (16).

Several basic and clinical studies have found that the radiotherapy dose (fractionation scheme) is closely related to the therapeutic effects on tumors. Riva et al. found that increasing the radiotherapy dose could upregulate the CD8^+^ T cell/regulatory T cell ratio in tumors, thereby inhibiting tumor cell proliferation and prolonging the survival of mice (17). Other researchers have discovered that the occurrence of ICD in breast cancer is related to the radiation dose, and the expression of the ICD marker CRT is dose-dependent (18). However, the effects of high-dose radiotherapy on NPC and the types of immune responses remain unclear. Therefore, this study aims to investigate the effects of high-dose radiation on apoptosis, cell cycle, and ICD in NPC cells, as well as the impact of irradiated NPC on the immunophenotype of DCs. This will provide important insights into the immunological functions of DCs, immune response, and clinical protocols for NPC radiotherapy.

## 2 Materials and methods

### 2.1 Cell culture

The 5-8F human NPC cells were acquired from Fenghbio Corporation (Hunan, China). The culture medium for 5-8F cells was RPMI 1640 (Gibco, Grand Island, NY). To enhance medium, 10% FBS (Biological Industries) fetal bovine serum (FBS; Biological Industries, Beit Hamek, Israel) and 100 IU/ml penicillin/streptomycin (Gibco) were added. When the cell density reaches 80–90% confluence, the cells are digested with trypsin, passaged, and continuously cultured in a 37°C, 5% CO_2_ incubator. When 5-8F cells reach the logarithmic growth phase, the old medium in the culture flask is discarded, and 5 mL of fresh complete medium is added. The cells are then placed in an irradiation device (RS-2000 system) and exposed to X-rays at 160 kV and 24 mA for 0, 109, or 543 seconds, corresponding to radiation doses of 0, 2, or 10 Gy, respectively.

### 2.2 Preparation of DC

Human peripheral blood monocytes were used to create DCs, as previously reported (19, 20). Following informed consent and approval by Guizhou Medical University’s Animal and Medicine Ethical Committee (#2020162, 2020/4/9), whole human blood was drawn from healthy, unmedicated donors in accordance with the Declaration of Helsinki’s guidelines. Regardless of age or sex, donors were chosen at random. Recombinant human granulo-cyte-macrophage colony-stimulating factor (rhGM-CSF) and recombinant human interleukin (IL)-4 (rhIL-4) (both from Peprotech, Cranbury, NY) were added to T-75 flasks containing complete RPMI-1640 medium after CD14+ monocytes from human peripheral blood were isolated using cocktail immune-magnetic beads (Miltenyi Biotec). For 5 days, these cells were kept at 37°C in order to induce immature DCs (imDCs).

### 2.3 Co-culture of NPC cells with imDCs

Collect imDCs and adjust the cell density to 1×10^6^ cells/mL using 1640 medium supplemented with 20% FBS for subsequent use. Retain the 5-8F cells that have been irradiated for 48 hours in 6-well plates along with their conditioned medium. Place the Transwell upper chambers into the 6-well plates containing the 5-8F cells, and add 2 mL of the imDC suspension (a total of 2×10^6^ imDCs). The experimental groups are as follows: 1) imDCs group: imDCs cultured in suspension under normal conditions. 2) imDCs + 0 Gy group: co-culture of imDCs with non-irradiated 5-8F cells. 3) imDCs + 2 Gy group**: co-culture of imDCs with 5-8F cells irradiated at 2 Gy. 4) imDCs + 10 Gy group**: co-culture of imDCs with 5-8F cells irradiated at 10 Gy. The negative control group consists of 2 mL of the imDC suspension and 3 mL of PBS. All groups are cultured at 37°C in a 5% CO_2_ incubator.

### 2.4 Apoptosis analysis

Using the FITC-Annexin V/PI double staining kit (Meilunbio) and flow cytometry, apoptosis was identified. In brief, after irradiation as above, 5-8F cells were centrifuged and then resuspended in 100 μl of binding buffer that contained 5 μl of Annexin V and 5 μl of PI. Following a 15-minute dark incubation period at room temperature, the cells were resuspended in 400 μl of binding buffer and subjected to analysis using a BD FACSCanto™ Flow Cytometer and FlowJo software version 10. Annexin V-positive cells with FITC labels were identified as apoptotic cells.

### 2.5 Reverse transcription-polymerase chain reaction

TRIzol (Invitrogen, Carlsbad, CA) was used to extract the cells’ total RNA in accordance with established procedures (21). To assess the quantity and quality of the extracted total RNA, a NanoDrop Ultramicro spectrophotometer (ThermoFirsher Scientific, Waltham, MA) was used. Using a Fast-king gDNA Dispelling RT Supermix (Tiangen, Beijing), 1 μg of total RNA/sample was reverse-transcribed to cDNA based on the results. Next, a SYBR Premix Ex Taq kit (Takara, Tokyo, Japan) was used to conduct real-time PCR. **Table 1** lists the precise primers that were utilized (22). In all cases, *GAPDH* served as the internal reference gene. The ΔΔCT method was then used to determine the mRNA expression levels of each gene of interest (23).

**Table 1:** Primers used in this study.

Gene	Primer sequences (5’ → 3’)
*GAPDH*	F: GACCTGACCTGCCGTCTA
	R: AGGAGTGGGTGTCGCTGT
*BAK1*	F: GGACGACATCAACCGACGCTATG
	R: AACAGGCTGGTGGCAATCTTGG
*CRT*	F: AGATAAAGGTTTGCAGACAAGC
	R: CATGTCTGTCTGGTCCAAACTA
*Casp-3*	F: GGAACAAATGGACCTGTTGAC
	R: CTCAATGCCACAGTCCAGTTC
*HMGB-1*	F: AAATGAAAACCTATATCCCTCCC
	R: GGGCGATACTCAGAGCAGAAG
*HSP70A1A*	F: GACTCCCGTTGTCCCAAG
	R: CGGTTCCCTGCTCTCTGT
*CD80*	F: GTGGTCACAATGTTTCTGTTGA
	R: GTTCTTGTACTCGGGCCATATA
*CD86*	F: TGCTCATCTATACACGGTTACC
	R: TGCATAACACCATCATACTCGA
*HLA-DR*	F: CCAGAGACTACAGAGAATGTGG
	R: TTGATGATGAAGATGGTCCCAA
*CCR5*	F: GCAGCTCTCATTTTCCATACAG
	R: GACACCGAAGCAGAGTTTTTAG
*CCR7*	F: AGACCATGACCGATACCTACC
	R: GCAAAAGTGGACACCGAAGA
*CD11c*	F: AGCAGCCACGAACAATTCAC
	R: GAGACCTCCACATCCATCCA

### 2.6 Western blot

The BCA protein assay kit (Solarbio) was used to quantify the amount of total protein recovered from 5-8F cells using RIPA cell lysis solution (Solarbio) enhanced with a protease inhibitor cocktail (Sigma). After being separated on an SDS-PAGE gel, 20 μg of total protein from each sample was deposited onto a 0.45-μm nitrocellulose membrane (Millipore). HMGB1 (D260488; BBI, Shanghai, China), GAPDH (10494-1-AP; Protein-tech, Rosemont, USA), CRT (AB92516; Abcam, Cambridge, UK), and HSP70 (AB181606, Abcam, Cambridge, UK) were the main antibodies utilized. The ECL detection method identified the immunolabeled proteins. ImageJ software (NIH, Bethesda, MD) was used to quantify the protein bands.

### 2.7 Enzyme-linked immunosorbent assay

After collecting the cell culture supernatants of 5-8F, they were centrifuged for five minutes at 1200 rpm. After being moved into fresh EP tubes, the supernatants were thoroughly combined. Following the manufacturer’s instructions, ELISA kits (Zikerbio, Shenzhen, China) were used to measure the amounts of human HSP70, HMGB1, CRT, IL-6, IL-12p70, TNF-α, IL-10, and IL-4 in the culture medium.

### 2.8 Statistical analyses

The mean ± SD of at least three separate experiments is used to express all data. Every piece of data was examined to make sure it met the homogeneity assumptions. A Student’s *t*-test was used to compare the treatment groups statistically. Multiple group comparisons were conducted using one-way analysis of variance (One-way ANOVA). A *p*-value of less than 0.05 was deemed significant. Prism software (v.7.0, GraphPad, San Diego, CA) was used for all data analyses, whereas picture J program was used for picture analysis.

## 3 Results

### 3.1 High-dose radiation promoted apoptosis among 5-8F cells

**Figure 1:**
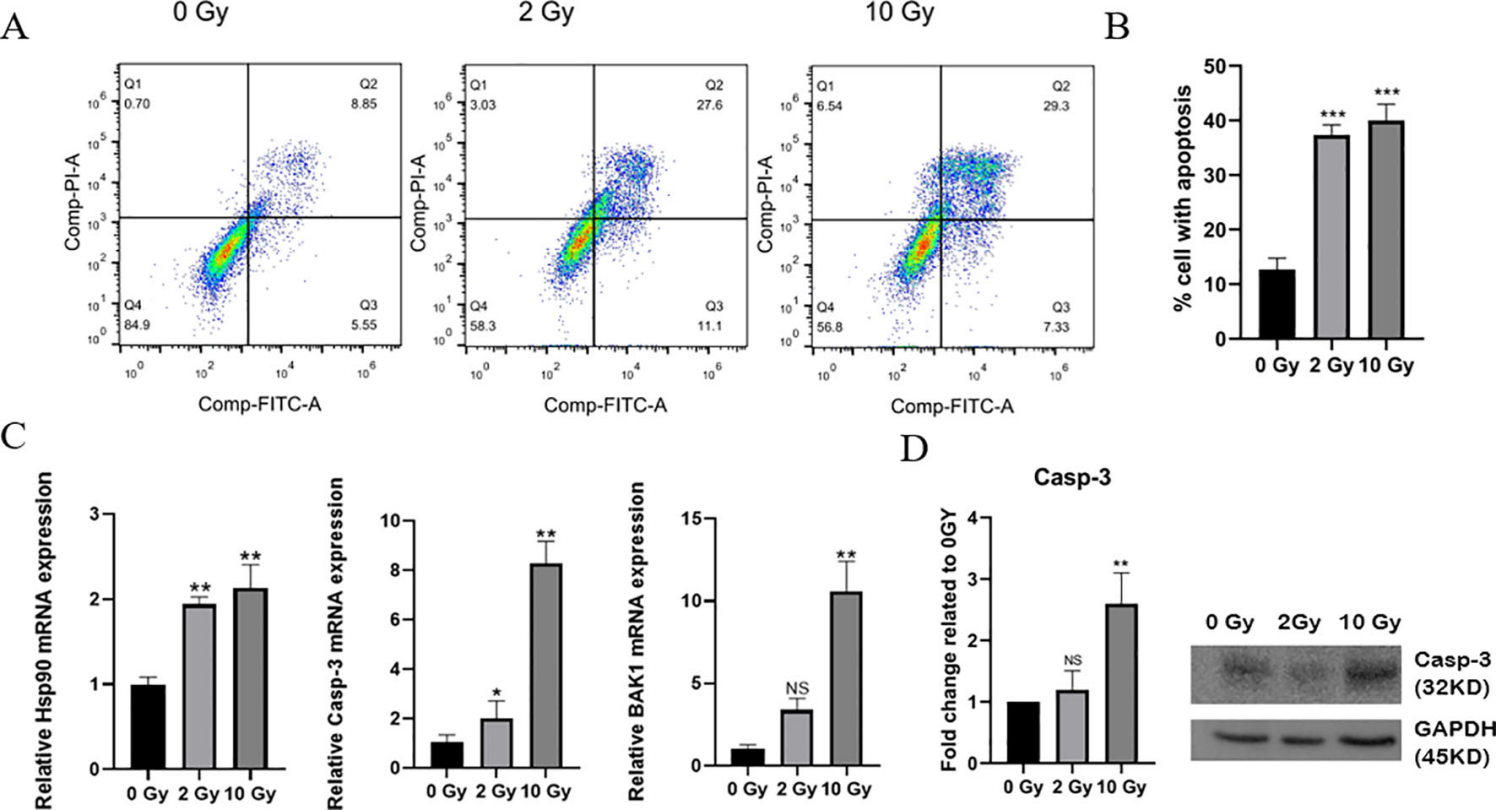
Apoptosis in irradiated 5-8F cells. **(A)** Apoptosis levels in cells after 0, 2, or 10 Gy exposures was analyzed by flow cytometry. **(B)** Statistics of the proportion of apoptotic cells. **(C)** QPCR was used to detect the mRNA expression levels of HSP90, Casp-3, and BAK1 in 5-8F cells after exposure to different radiation doses. **(D)** Western Bolting was used to assess the expression of the apoptotic protein Caspase-3 in 5-8F cells following various radiation doses. ^*^ indicates a statistically significant difference compared to the 0 Gy group. NS *p* > 0.05, ^*^, ^**^, ^***^ *p* < 0.05, < 0.01, < 0.001.

Research has shown that radiation can cause single- and double-strand breaks in DNA and induce the release of radiation-induced reactive oxygen species, ultimately leading to apoptosis (24, 25). To study the effects of high-dose radiation on apoptosis in NPC cells, flow cytometry was used to assess apoptosis in 5-8F cells 48 hours after irradiated with doses of 0, 2, or 10 Gy. The results showed that the number of apoptotic cells increased significantly following 10 Gy irradiation (**Figures 1A, B**), with the apoptosis rate reaching 40%, including an early apoptosis rate of 28.3% and a late apoptosis rate of 11.7%. Furthermore, the mRNA expression levels of HSP90, Casp-3, and BAK1 gradually increased with the radiation dose (**Figure 1C**). The WB results revealed that the expression of Casp-3 protein significantly increased in 5-8F cells after 10 Gy irradiation, consistent with the qPCR findings (**Figure 1D**). Thus, high-dose radiation can induce more apoptosis in NPC cells, making it a more effective means of killing these cells.

### 3.2 Immunogenic cell death of 5-8F cells induced by radiation

**Figure 2:**
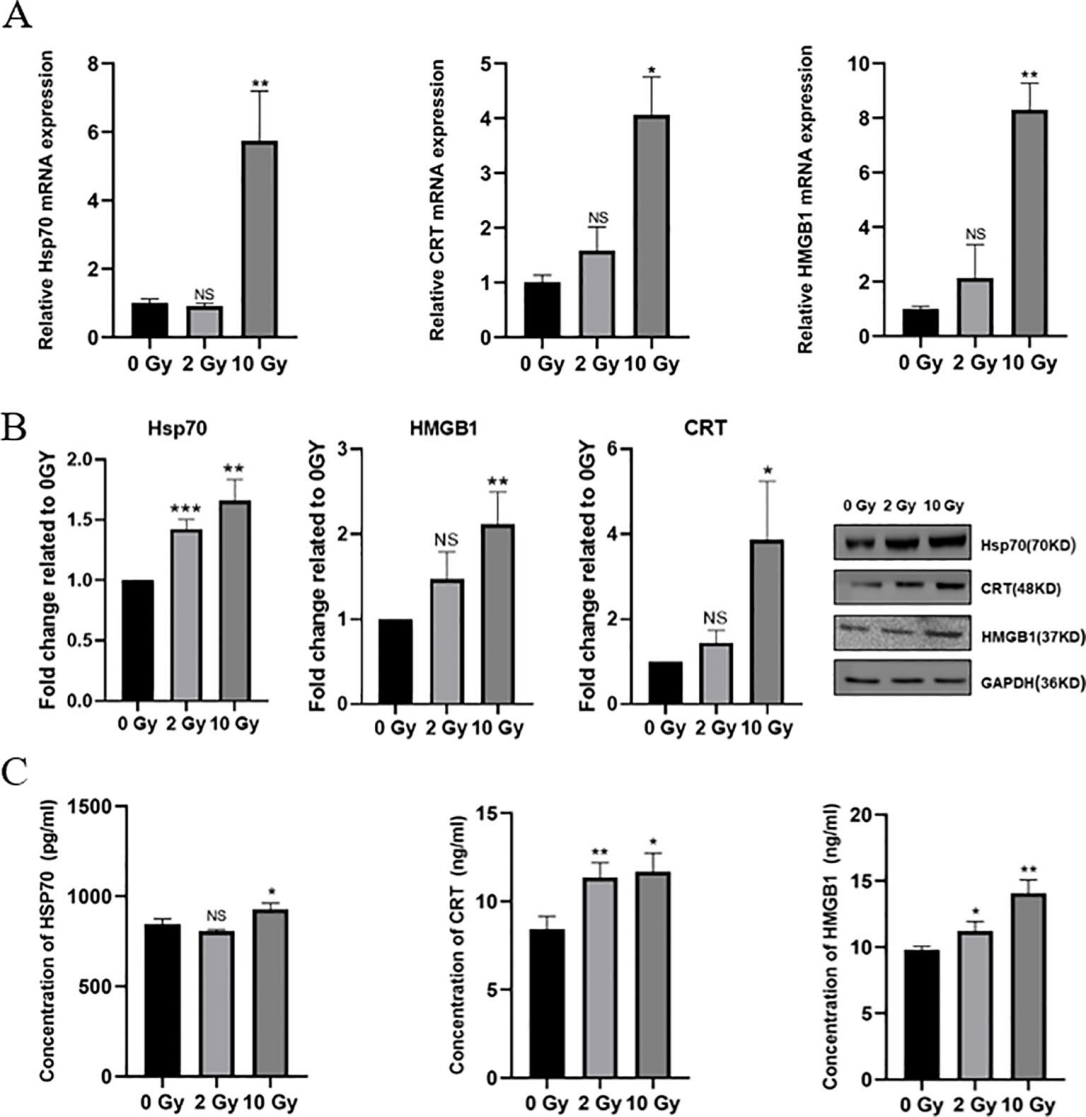
Select ICD-related proteins in irradiated 5-8F cells. **(A)** mRNA expression of HSP70 and CRT. **(B)** Protein expression levels of HSP70 and CRT. **(C)** Concentration of HSP70, CRT and HMGB1 in conditioned medium. NS *p* > 0.05, ^*^, ^**^, ^***^ *p* < 0.05, < 0.01, < 0.001.

Radiation damages tumor cells, causing dying tumor cells to release DAMPs, which in turn induces ICD in tumor cells. To investigate the effect of high-dose radiation on ICD in NPC cells, we first used qPCR to measure the mRNA expression levels of HSP70, CRT and HMGB1. The results showed that the expressions of HSP70, CRT and HMGB1 were significantly upregulated after high-dose radiation, with notable statistical significance (**Figure 2A**). Western blotting results indicated that as the radiation dose increased, the expression of ICD-related proteins in 5-8F cells also gradually increased (**Figure 2B**). After the irradiation treatments, it was also seen that the levels of HSP70, CRT and HMGB1 in the 5-8F cell culture medium increased in tandem with the levels of radiation employed (**Figure 2C**). Therefore, high-dose radiation can upregulate the expression of ICD-related proteins in NPC 5-8F cells, thereby inducing ICD in these cells.

### 3.3 High-dose radiation impact on 5-8F cell secretion of inflammatory cytokines

**Figure 3:**
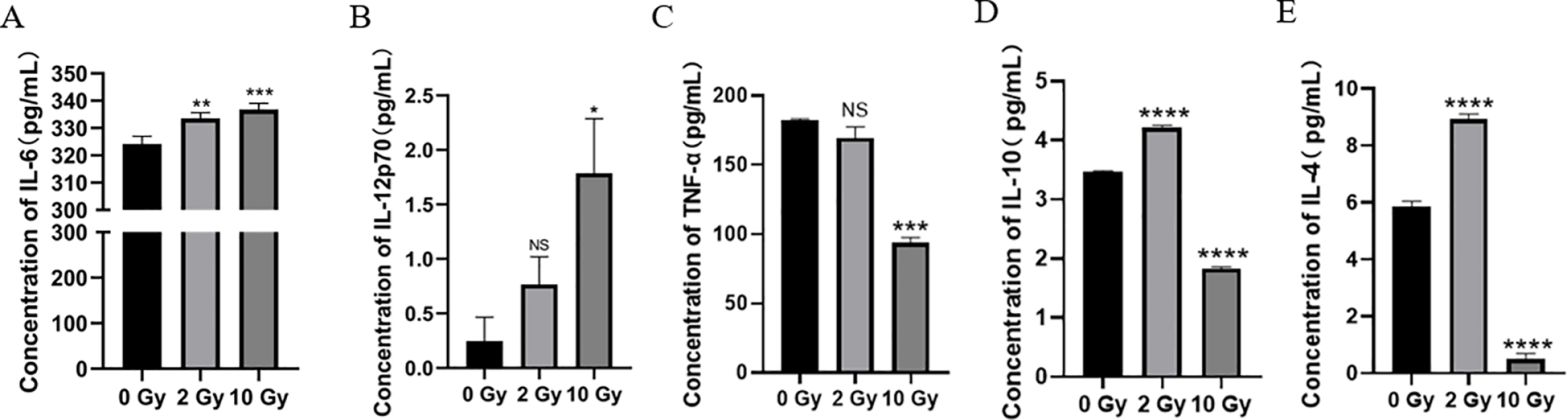
The effect of high dose radiation on secretion of inflammatory cytokines in NPC cells. The concentration of **(A)** IL-6, IL-12p70 **(B)**, TNF-α **(C)**, IL-10 **(D)** and IL-4 **(E)** in conditioned medium. NS *p* > 0.05, *, **, ***, **** *p* < 0.05, < 0.01, < 0.001, < 0.0001.

Radiation can alter the levels of various cytokines in the tumor microenvironment (TME). To study the effect of high-dose radiation on the secretion of inflammatory cytokines by NPC cells, we used ELISA to measure the levels of inflammatory cytokines in the culture supernatant of 5-8F cells after irradiation. The results showed (**Figures 3A, B**) that Compared to the 0 Gy group, the concentrations of IL-6 and IL-12p70 in the supernatant of the 10 Gy group were significantly increased (*p* < 0.05). The secretion of pro-inflammatory cytokines was dose-dependent. Thus, high-dose radiation promotes the secretion of more IL-6 and IL-12p70 by 5-8F cells. Additionally, our results also showed (**Figures 3C–E**) that after 10 Gy irradiation, the levels of TNF-α, IL-10, and IL-4 all decreased significantly. Therefore, high-dose radiation can reduce the secretion of TNF-α, IL-10, and IL-4 by 5-8F cells.

### 3.4 The impact of high-dose irradiated NPC cells on immature DCs

**Figure 4:**
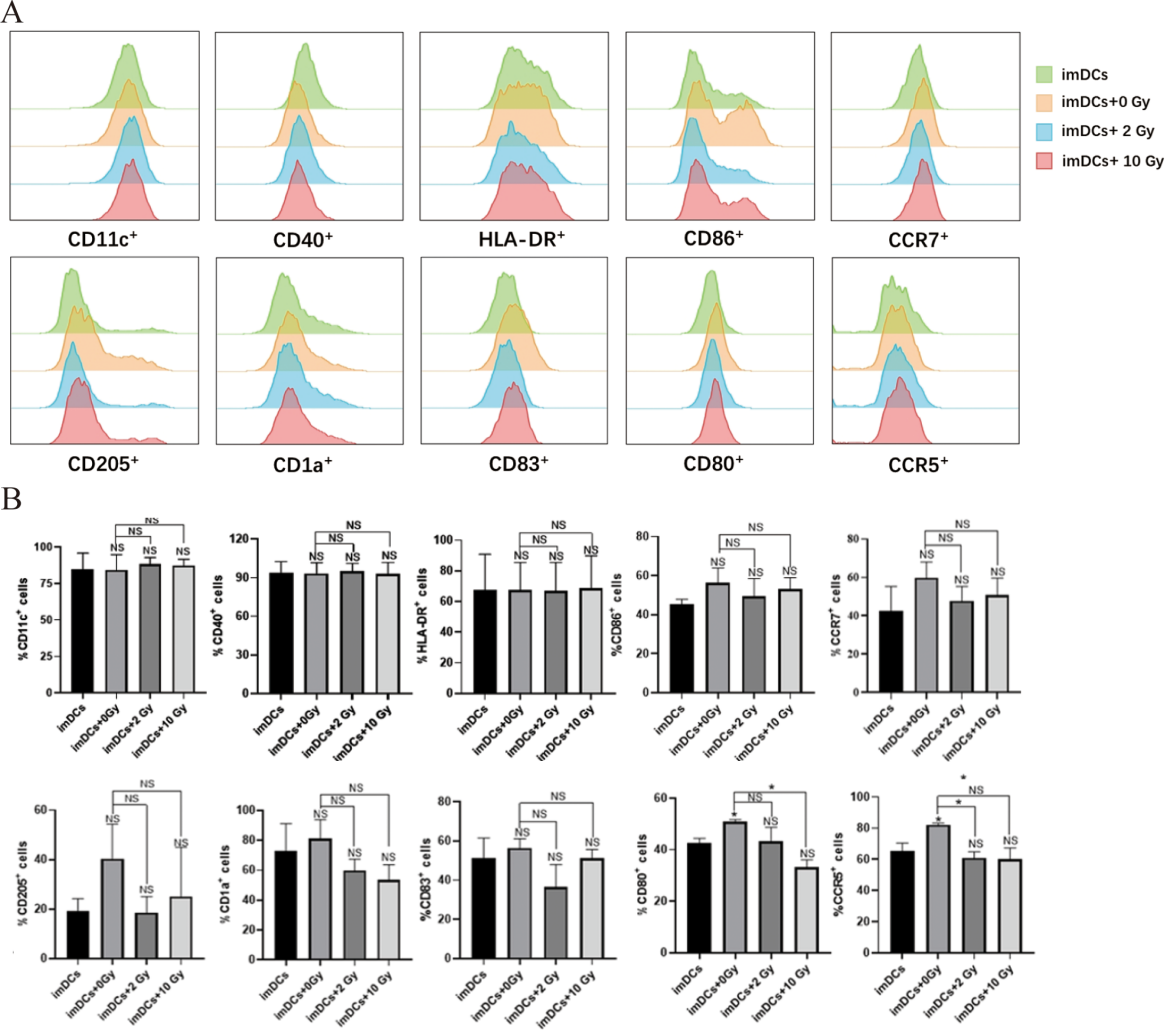
The protein expression of surface molecules in immature dendritic cells cocultured with NPC cell for 48 h. **(A)** Flow cytometry was used to detect the expression of surface molecules on DCs, including CD11c, CD40, HLA-DR, CD86, CCR7, CD205, CD1a, CD83, CD80 and CCR5. **(B)** In the statistical graph, imDCs = control group (DC without conditioned 5-8F cell media). imDCs + 0 Gy = DC cultured with media of 5-8F cells without radiation treatment. Various radiation doses used to treat 5-8F cells prior to isolation of their media to then treat DC cells arenoted: 2, and 10 Gy. NS *p* > 0.05, *p < 0.05.

**Figure 5:**
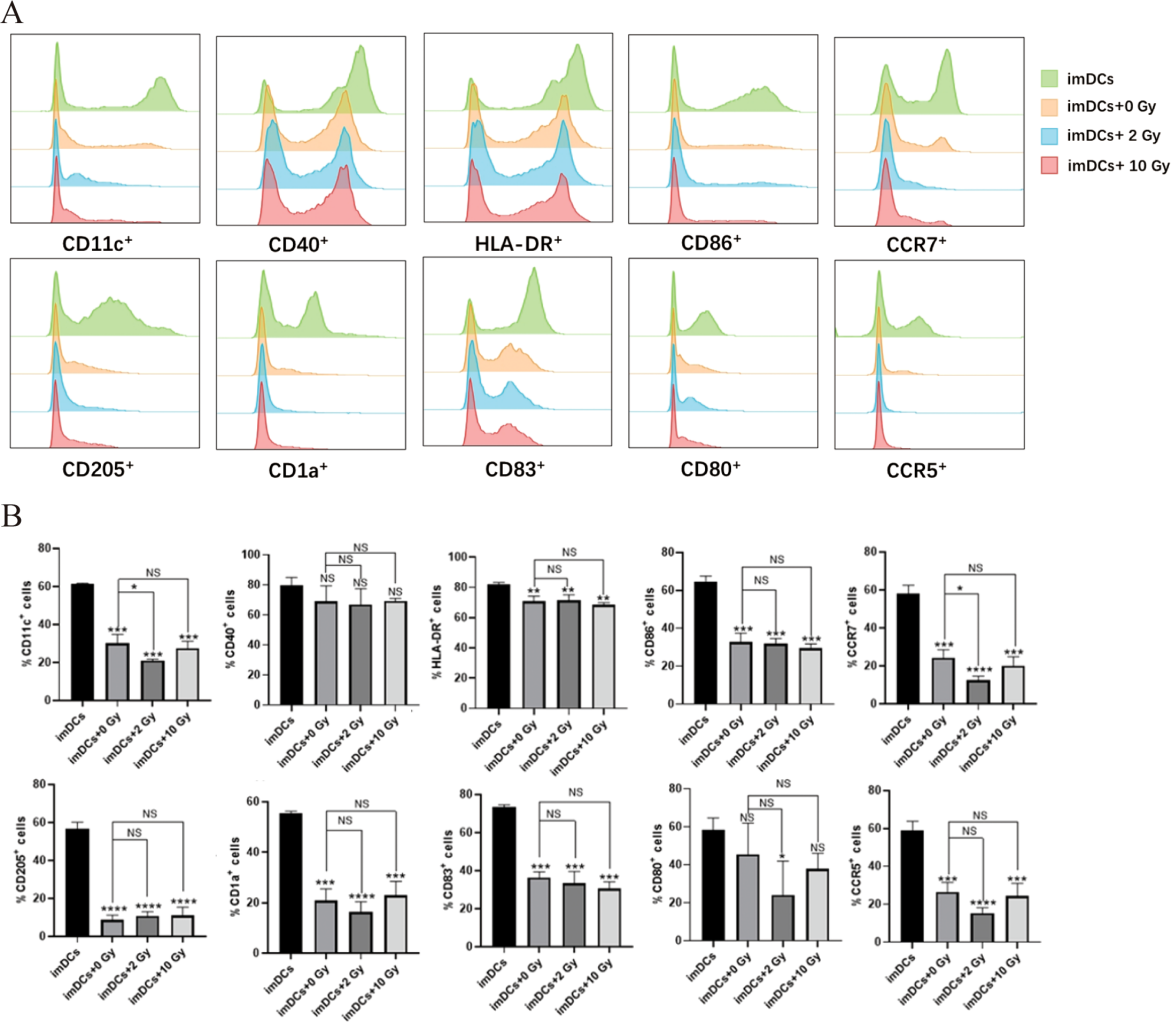
The protein expression of surface molecules in immature dendritic cells cocultured with NPC cell for 72 h. **(A)** Flow cytometry was used to detect the expression of surface molecules on DCs, including CD11c, CD40, HLA-DR, CD86, CCR7, CD205, CD1a, CD83, CD80 and CCR5. **(B)** Relative statistical graph. NS p > 0.05, *, **, ***, **** *p* < 0.05, < 0.01, < 0.001, < 0.0001.

Research has shown that radiation-induced ICD in tumor cells, along with changes in the TME, can activate antigen-presenting cells and enhance their immune recognition and antigen-presenting functions. This promotes the recruitment of effector T cells, strengthening the immune response to kill tumor cells (7). As the most potent APCs in the body, DCs play a critical role in antitumor immune responses. Therefore, in this study, we co-cultured imDCs with irradiated NPC cells for 48 hours and used flow cytometry to assess the expression of surface molecules on the co-cultured DCs. The results (**Figure 4**) indicated that after 48 hours of co-culture, there were no significant changes in the expression of surface molecules CD11c, CD40, HLA-DR, CD86, CCR7, CD205, CD1a, CD83, CD80 and CCR5. We then performed flow cytometry again to assess the surface molecules on DCs after 72 hours of co-culture (**Figure 5**). The results showed that, compared to the 0 Gy group, co-culture with 2 Gy or 10 Gy irradiated NPC cells for 72 hours led to a significant decrease in the expression of CD80, CD86, CD11c, CD40, HLA-DR, CCR5, CCR7, CD205, CD1a, and CD83 on DCs (*P*<0.01). This indicates that prolonged co-culture with NPC cells following high-dose radiation inhibits the expression of surface molecules on DCs.

### 3.5 The expression levels of surface molecules on DCs after short-term co-culture with NPC cells

**Figure 6:**
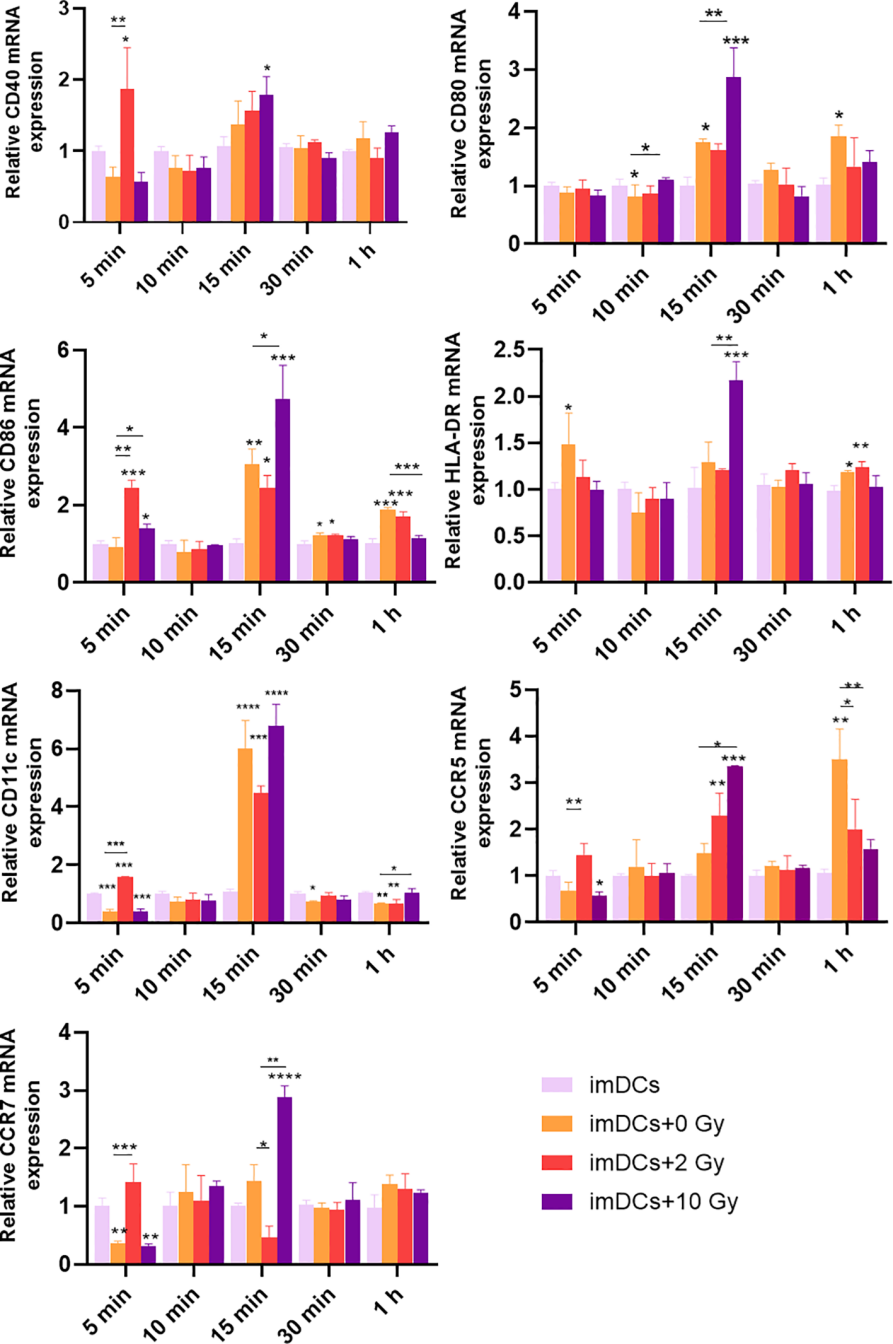
Expression levels of genes for DC surface molecules after DC treatment with conditioned media of irradiated NPC cell for a short time. The mRNA expression levels of CD40, CD80, CD86, HLA-DR, CD11c, CCR5, and CCR7 after co-culture for 5 min, 10 min, 15 min, 30 min, and 1 h. NS *p* > 0.05, ^*^, ^**^, ^***^ *p* < 0.05, < 0.01, < 0.001.

Recent studies have shown that the innate immune response may be activated within minutes to hours after antigen presentation (26). Therefore, in this study, qPCR was used to examine the transcriptional levels of surface molecules on DCs after co-culturing for 5 minutes, 10 minutes, 15 minutes, 30 minutes, and 1 hour. The results (**Figure 6**) demonstrated that after 5 minutes of co-culture, the expression of CD86, CD40, CD11c, CCR5, and CCR7 was significantly upregulated in DCs co-cultured with 5-8F cells irradiated with 2 Gy, compared to the imDCs + 0 Gy group. At 15 minutes of co-culture, compared to the imDCs control group, there were no significant differences in the expression of CD80, CD86, CD40, HLA-DR, CD11c, and CCR5 in DCs co-cultured with 5-8F cells irradiated with 2 Gy. However, in DCs co-cultured with 5-8F cells irradiated with 10 Gy, the expression of CD80, CD86, HLA-DR, CCR5, and CCR7 was significantly upregulated. In conclusion, co-culture times of 5 minutes, 10 minutes, 30 minutes, and 1 hour had minimal effects on the expression of surface molecules in DCs. However, the most significant impact was observed at 15 minutes, during which the expression of surface molecules on DCs co-cultured with 5-8F cells irradiated with a high dose of radiation was significantly increased compared to the 0 Gy control group.

## 4 Discussion

Radiotherapy is the primary treatment for the majority of cancer patients (27). NPC, an epithelial carcinoma originating from the mucosal lining of the nasopharynx, exhibits high sensitivity to radiotherapy. However, due to the anatomical constraints of surrounding organs, the conventional fractionation regimen for NPC still employs standard doses (1.8-2.2 Gy/fraction) (28). Due to the detachment of dead cells after radiation exposure, the contact inhibition among the remaining cells disappears, leading to accelerated proliferation of the surviving cells (29). This ultimately manifests as an increase in cell viability, which is consistent with the concept of cellular repopulation in the 4R’s of radiobiology. Therefore, the subsequent experimental groups were defined as follows: the non-radiation group (0 Gy), the conventional-dose radiation group (2 Gy), and the high-dose radiation group (10 Gy). With advances in radiotherapy techniques, high-dose radiation has been applied clinically, and localized high-dose radiotherapy can activate DCs to induce antigen-specific T-cell immune responses (30). However, studies on the effects of high-dose radiation on NPC are limited, and there are few reports on how NPC cells exposed to high-dose radiation affect DCs. Thus, our study investigated the effects of high-dose radiation on NPC in terms of cell apoptosis and ICD. We also explored the impact of NPC cells treated with high-dose radiation on DCs. This research will help to further understand the sensitivity of NPC to different radiation doses and the changes in DC immune phenotypes during radiotherapy, providing theoretical support for the clinical treatment strategies of NPC.

The clinical efficacy of radiotherapy is closely related to its induction of autophagy, apoptosis, and the abscopal effect in tumor cells (24, 25, 31), with apoptosis being the primary form of cell death following radiotherapy (32). Our study found that the number of apoptotic NPC cells significantly increased after high-dose radiation (**Figures 1A, B**). Furthermore, the expression of apoptosis-related genes was then examined (**Figure 1C**). Our results demonstrated that the expression of HSP90, Casp-3, BAK1, and the Casp-3 protein all increased after high-dose radiation, promoting apoptosis in NPC cells. These findings are consistent with the flow cytometry results showing increased apoptosis. HSP90 is a marker of ICD, and its increased exposure on the cell surface can promote the maturation of DCs, thereby inducing an antitumor immune response (33, 34). This suggested that high-dose radiotherapy may induce DC-mediated immune response in NPC.

Radiotherapy damages tumor cells, causing dying tumor cells to release DAMPs, which can induce ICD and thereby activate APCs, enhancing the immune system’s response to tumor cells (35). These DAMP components interact with phagocytic receptors, purinergic receptors, and pattern recognition receptors (PRRs) on the surface of innate immune cells (36). Additionally, they act as activators, stimulating APCs to present antigens on MHC-I and MHC-II molecules to T cells, thereby effectively facilitating antigen presentation and mobilizing adaptive immune responses (37). In this study, we further investigated the effect of high-dose radiation on the expression of ICD-related molecules in 5-8F NPC cells. This corresponds with our findings, where the expression of HMGB1 and apoptosis in NPC cells both peaked at 10 Gy (**Figure 2**). Thus, high-dose radiation can induce ICD in 5-8F NPC cells, enhancing the immunogenicity of NPC and may promote the recruitment and maturation of DCs.

Cytokines play a crucial role in antitumor immunity (38), which is why this study investigates the levels of pro-inflammatory cytokines (IL-6 and IL-12p70) and anti-inflammatory cytokines (TNF-α, IL-10, and IL-4) in the supernatant of 5-8F cell cultures (**Figure 3**). The pro-inflammatory cytokine IL-6 is not only an essential inflammatory signal for the migration of mature DCs to lymph nodes (39) but also promotes the specific differentiation of CD8^+^ T cells into cytotoxic T cells (40). IL-12 stimulates the proliferation and activation of CD8^+^ T lymphocytes, thereby enhancing the antitumor immune response (41). After high-dose radiation, the levels of pro-inflammatory cytokines in the 5-8F cell culture supernatant were significantly higher than those in cells treated with 2Gy radiation, facilitating the antigen presentation by DCs to T cells and promoting antitumor immune responses. Research by Yu et al. has shown that TNF-α levels in the tumor tissues of NPC patients are associated with poor prognosis (42). High concentrations of IL-10 in NPC patients promote the proliferation of NPC cells and inhibit the antigen presentation by DCs and the function of cytotoxic T cells (43). Studies have shown that IL-4 mediates tumor cell proliferation and metastasis in gastric and colorectal cancers (44). Our study found that the levels of anti-inflammatory cytokines in the 5-8F cell culture supernatant were significantly reduced after high-dose radiation. Furthermore, high-dose radiation promotes the formation of an immunogenic TME in NPC, facilitating the maturation and differentiation of DCs and T cells, thereby enhancing the antitumor immune response.

Radiotherapy induces ICD in tumor cells and alters the TME, which can activate APCs and enhance their immune recognition and antigen-presenting functions (45). This facilitates the recruitment of effector T cells, boosting the immune response to ultimately kill tumor cells (18, 46). In recent years, the widespread application of immunotherapy has significantly improved the prognosis of NPC patients (47). However, radiotherapy remains the cornerstone of NPC treatment. From the perspective of radioimmunology, the key factors in radioimmunology include the types of antigens released, the function of APCs, and the resulting immune response. These factors can either contribute to an immunosuppressive tumor microenvironment or break immune tolerance to the tumor, thereby initiating a specific anti-tumor immune response and exerting an anti-tumor effect (48).

As the most potent APCs in the body, DCs upregulate the expression of costimulatory molecules such as CD80, CD86, CD40, and the chemokine receptor CCR7 upon stimulation with tumor antigens (49, 50). In this study, imDCs were co-cultured with NPC cells post-radiation to assess the expression of surface molecules on DCs. The results (**Figures 4**, **5**) showed that, compared to normally cultured imDCs, the expression of surface molecules on imDCs was reduced after long-term co-culture (48 h or 72 h) with NPC cells, whether irradiated or not. This may be due to the long-term action of other immunosuppressive cytokines (such as VEGF, TGF-β, and lactate) released by NPC cells, leading to the downregulation of surface molecules such as CD80, CD86, and CD11c on imDCs (51, 52). Studies have suggested that DCs can quickly phagocytose antigens and upregulate the expression of related proteins (53, 54). Consistently, our results showed that when the co-culture time was shortened, the inhibition of surface molecule expression on imDCs co-cultured with NPC cells was alleviated. The expression of CD80, CD86, and HLA-DR molecules increases as DCs mature. Notably (**Figure 6**), when the co-culture time was reduced to 15 minutes, the expression of surface molecules on imDCs co-cultured with NPC cells post-high-dose radiation significantly increased. It can be inferred that at a co-culture duration of 15 minutes, DCs are stimulated by tumor antigens and undergo maturation, which may facilitate the activation of cytotoxic T cells and enhance the anti-tumor immune response. This suggests that DC recognition and response to NPC tumor antigens occur within a short time frame, and prolonged co-culture leads to inhibition of DC maturation due to other immunosuppressive factors. This highlights the importance of the timing of immunotherapy in combination with radiotherapy.

Many studies have noted that radiation exposure alters the function and cytokine secretion profile of DCs. Yu N et al. found that exposure to 0.2 Gy X-rays significantly enhanced the migration ability of DCs and their IL-12 production, while also upregulating CCR7 expression (55). However, Merrick et al. reported that irradiated DCs produced less IL-12 and exhibited impaired activation of naïve T cells compared to non-irradiated DCs (56). These findings suggest that the effects of radiation on the antigen-presenting function of DCs remain controversial. Our study demonstrates that in addition to directly killing NPC cells, hypofractionated ionizing radiation therapy can induce the differentiation of imDCs into mDCs, thereby initiating a specific anti-tumor immune response. These findings have important implications for a deeper understanding of clinical treatment strategies for NPC. The limitation of this study is that it only investigated, *in vitro*, the effect of NPC cells on DC maturation (surface molecule expression) after radiotherapy. However, *in vitro* experiments have shown that co-culture duration significantly influences DC maturation, which is likely critical for the activation of anti-tumor immune responses *in vivo* as well. Additionally, this study did not further explore the post-radiotherapy immune status of NPC patients in a clinical setting.

## 5 Conclusion

In conclusion, compared to conventional radiation doses (2 Gy), high-dose radiation (10 Gy) more effectively induces apoptosis, cell cycle arrest, and immunogenic cell death in NPC 5-8F cells. Additionally, short-term co-culture with NPC 5-8F cells exposed to high-dose radiation can induce DC maturation. It appears that NPC cells significantly influence the maturation of DCs and after high-dose radiation therapy.

## Data Availability

The raw data supporting the conclusions of this article will be made available by the authors, without undue reservation.

## References

[1] Engbang JP Njifou A Tjomb T Mpessa M Njock L. Prognosis and survival of nasopharyngeal cancer in Cameroon. Int J Otolaryngol Head Neck Surgery. (2021) 10:6–19. doi: 10.4236/ijohns.2021.101002

[2] Guan S Wei J Huang L Wu L. Chemotherapy and chemo-resistance in nasopharyngeal carcinoma. Eur J Medicinal Chem. (2020) 207:112758. doi: 10.1016/j.ejmech.2020.112758 32858472

[3] Huang H Yao Y Deng X Huang Z Chen Y Wang Z. Immunotherapy for nasopharyngeal carcinoma: Current status and prospects (Review). Int J Oncol. (2023) 63:97. doi: 10.3892/ijo.2023.5545 37417358 PMC10367053

[4] Ding X Zhang W-J You R Zou X Wang Z-Q Ouyang Y-F. Camrelizumab plus apatinib in patients with recurrent or metastatic nasopharyngeal carcinoma: an open-label, single-arm, phase II study. J Clin Oncol. (2023) 41:2571–82. doi: 10.1200/JCO.22.01450 PMC10414735 36735896

[5] Lee AW Poon YF Foo W Law SC Cheung FK Chan DK. Retrospective analysis of 5037 patients with nasopharyngeal carcinoma treated during 1976-1985: overall survival and patterns of failure. Int J Radiat Oncol Biol Physics. (1992) 23:261–70. doi: 10.1016/0360-3016(92)90740-9 1587745

[6] Santivasi WL Xia F. Ionizing radiation-induced DNA damage, response, and repair. Antioxid Redox Signaling. (2014) 21:251–9. doi: 10.1089/ars.2013.5668 24180216

[7] Portella L Scala S. Ionizing radiation effects on the tumor microenvironment. Semin Oncol. (2019) 46:254–60. doi: 10.1053/j.seminoncol.2019.07.003 31383368

[8] Asna N Livoff A Batash R Debbi R Schaffer P Rivkind T. Radiation therapy and immunotherapy-a potential combination in cancer treatment. Curr Oncol (Toronto Ont). (2018) 25:e454–e60. doi: 10.3747/co.25.4002 PMC6209565 30464697

[9] Hou Y Liang H Rao E Zheng W Huang X Deng L. Non-canonical NF-κB antagonizes STING sensor-mediated DNA sensing in radiotherapy. Immunity. (2018) 49:490–503.e4. doi: 10.1016/j.immuni.2018.07.008 30170810 PMC6775781

[10] Bedini N Cicchetti A Palorini F Magnani T Zuco V Pennati M. Evaluation of mediators associated with the inflammatory response in prostate cancer patients undergoing radiotherapy. Dis Markers. (2018) 2018:9128128. doi: 10.1155/2018/9128128 29682101 PMC5845513

[11] Lugade AA Sorensen EW Gerber SA Moran JP Frelinger JG Lord EM. Radiation-induced IFN-gamma production within the tumor microenvironment influences antitumor immunity. J Immunol (Baltimore Md: 1950). (2008) 180:3132–9. doi: 10.4049/jimmunol.180.5.3132 18292536

[12] Zhou J Wang G Chen Y Wang H Hua Y Cai Z. Immunogenic cell death in cancer therapy: Present and emerging inducers. J Cell Mol Med. (2019) 23:4854–65. doi: 10.1111/jcmm.2019.23.issue-8 PMC6653385 31210425

[13] Rapoport BL Anderson R. Realizing the clinical potential of immunogenic cell death in cancer chemotherapy and radiotherapy. Int J Mol Sci. (2019) 20(4):959. doi: 10.3390/ijms20040959 30813267 PMC6412296

[14] Derer A Deloch L Rubner Y Fietkau R Frey B Gaipl US. Radio-immunotherapy-induced immunogenic cancer cells as basis for induction of systemic anti-tumor immune responses - pre-clinical evidence and ongoing clinical applications. Front Immunol. (2015) 6:505. doi: 10.3389/fimmu.2015.00505 26500646 PMC4597129

[15] Vaes RDW Hendriks LEL Vooijs M De Ruysscher D. Biomarkers of radiotherapy-induced immunogenic cell death. Cells. (2021) 10(4):930. doi: 10.3390/cells10040930 33920544 PMC8073519

[16] Yang D Chen Q Yang H Tracey KJ Bustin M Oppenheim JJ. High mobility group box-1 protein induces the migration and activation of human dendritic cells and acts as an alarmin. J Leukoc Biol. (2007) 81:59–66. doi: 10.1189/jlb.0306180 16966386

[17] Riva M Wouters R Nittner D Ceuster J Sterpin E Giovannoni R. Radiation dose-escalation and dose-fractionation modulate the immune microenvironment, cancer stem cells and vasculature in experimental high-grade gliomas. J Neurosurgical Sci. (2020) 67(1):55–65. doi: 10.23736/S0390-5616.20.05060-2 33056947

[18] Golden EB Frances D Pellicciotta I Demaria S Helen-Barcellos-Hoff M Formenti SC. Radiation fosters dose-dependent and chemotherapy-induced immunogenic cell death. Oncoimmunology. (2014) 3:e28518. doi: 10.4161/onci.28518 25071979 PMC4106151

[19] Xia H Zhang L Dai J Liu X Zhang X Zeng Z. Effect of selenium and peroxynitrite on immune function of immature dendritic cells in humans. Med Sci Monit. (2021) 27:e929004. doi: 10.12659/MSM.929004 33684094 PMC7953518

[20] Hu W Wang Y Chen J Yu P Tang F Hu Z. Regulation of biomaterial implantation-induced fibrin deposition to immunological functions of dendritic cells. Mater Today Bio. (2022) 14:100224. doi: 10.1016/j.mtbio.2022.100224 PMC8894278 35252832

[21] Chomczynski P. A reagent for the single-step simultaneous isolation of RNA, DNA and proteins from cell and tissue samples. Biotechniques. (1993) 15:532–4, 6-7. 7692896

[22] Zeng X Luo D Zhang S Cui Z Wang Y Chen J. High-dose radiation-induced immunogenic cell death of bladder cancer cells leads to dendritic cell activation. PloS One. (2024) 19:e0307024. doi: 10.1371/journal.pone.0307024 39231199 PMC11373825

[23] Livak KJ Schmittgen TD. Analysis of relative gene expression data using real-time quantitative PCR and the 2–ΔΔCT method. Methods. (2001) 25:402–8. doi: 10.1006/meth.2001.1262 11846609

[24] Gao L Zheng H Cai Q Wei L. Autophagy and tumour radiotherapy. Adv Exp Med Biol. (2020) 1207:375–87. doi: 10.1007/978-981-15-4272-5_25 32671760

[25] Guo L Zhang L Guan Y Li Y Zhang C Guo Q. *In vitro* studies of H520 cell cycle and apoptosis by anlotinib combined with radiotherapy. Thorac Cancer. (2021) 12:593–602. doi: 10.1111/1759-7714.13780 33438349 PMC7919126

[26] Voros O Panyi G Hajdu P. Immune synapse residency of orai1 alters ca(2+) response of T cells. Int J Mol Sci. (2021) 22(21):11514. doi: 10.3390/ijms222111514 34768945 PMC8583858

[27] Huang RX Zhou PK. DNA damage response signaling pathways and targets for radiotherapy sensitization in cancer. Signal Transduct Target Ther. (2020) 5:60. doi: 10.1038/s41392-020-0150-x 32355263 PMC7192953

[28] Chen YP Chan ATC Le QT Blanchard P Sun Y Ma J. Nasopharyngeal carcinoma. Lancet (London England). (2019) 394:64–80. doi: 10.1016/S0140-6736(19)30956-0 31178151

[29] Ruivo CF Adem B Silva M Melo SA. The biology of cancer exosomes: insights and new perspectives. Cancer Res. (2017) 77:6480–8. doi: 10.1158/0008-5472.CAN-17-0994 29162616

[30] Sato H Demaria S Ohno T. The role of radiotherapy in the age of immunotherapy. Japanese J Clin Oncol. (2021) 51:513–22. doi: 10.1093/jjco/hyaa268 PMC8012351 33561212

[31] Wilkins AC Patin EC Harrington KJ Melcher AA. The immunological consequences of radiation-induced DNA damage. J Pathol. (2019) 247:606–14. doi: 10.1002/path.2019.247.issue-5 30632153

[32] Gong L Zhang Y Liu C Zhang M Han S. Application of radiosensitizers in cancer radiotherapy. Int J Nanomed. (2021) 16:1083–102. doi: 10.2147/IJN.S290438 PMC7886779 33603370

[33] Zunino B Rubio-Patiño C Villa E Meynet O Proics E Cornille A. Hyperthermic intraperitoneal chemotherapy leads to an anticancer immune response via exposure of cell surface heat shock protein 90. Oncogene. (2016) 35:261–8. doi: 10.1038/onc.2015.82 25867070

[34] Ahmed A Tait SWG. Targeting immunogenic cell death in cancer. Mol Oncol. (2020) 14:2994–3006. doi: 10.1002/1878-0261.12851 33179413 PMC7718954

[35] Power R Lowery MA Reynolds JV Dunne MR. The cancer-immune set point in oesophageal cancer. Front Oncol. (2020) 10:891. doi: 10.3389/fonc.2020.00891 32582553 PMC7287212

[36] Denning NL Aziz M Gurien SD Wang P. DAMPs and NETs in sepsis. Front Immunol. (2019) 10:2536. doi: 10.3389/fimmu.2019.02536 31736963 PMC6831555

[37] Barker HE Paget JT Khan AA Harrington KJ. The tumour microenvironment after radiotherapy: mechanisms of resistance and recurrence. Nat Rev Cancer. (2015) 15:409–25. doi: 10.1038/nrc3958 PMC4896389 26105538

[38] Arnold KM Flynn NJ Raben A Romak L Yu Y Dicker AP. The impact of radiation on the tumor microenvironment: effect of dose and fractionation schedules. Cancer Growth Metastasis. (2018) 11:1179064418761639. doi: 10.1177/1179064418761639 29551910 PMC5846913

[39] Matsumura F Polz R Singh S Matsumura A Scheller J Yamashiro S. Investigation of fascin1, a marker of mature dendritic cells, reveals a new role for IL-6 signaling in CCR7-mediated chemotaxis. J Immunol (Baltimore Md: 1950). (2021) 207:938–49. doi: 10.4049/jimmunol.2000318 PMC8360331 34301846

[40] Okada M Kitahara M Kishimoto S Matsuda T Hirano T Kishimoto T. IL-6/BSF-2 functions as a killer helper factor in the *in vitro* induction of cytotoxic T cells. J Immunol (Baltimore Md: 1950). (1988) 141:1543–9. doi: 10.4049/jimmunol.141.5.1543 3261754

[41] Galdiero MR Marone G Mantovani A. Cancer inflammation and cytokines. Cold Spring Harbor Perspect Biol. (2018) 10. doi: 10.1101/cshperspect.a028662 PMC6071493 28778871

[42] Yu Y Ke L Xia WX Xiang Y Lv X Bu J. Elevated levels of TNF-α and decreased levels of CD68-positive macrophages in primary tumor tissues are unfavorable for the survival of patients with nasopharyngeal carcinoma. Technol Cancer Res Treat. (2019) 18:1533033819874807. doi: 10.1177/1533033819874807 31522611 PMC6747870

[43] Ren Y Yang J Li M Huang N Chen Y Wu X. Viral IL-10 promotes cell proliferation and cell cycle progression via JAK2/STAT3 signaling pathway in nasopharyngeal carcinoma cells. Biotechnol Appl Biochem. (2020) 67:929–38. doi: 10.1002/bab.v67.6 31737947

[44] Song X Traub B Shi J Kornmann M. Possible roles of interleukin-4 and -13 and their receptors in gastric and colon cancer. Int J Mol Sci. (2021) 22(2):727. doi: 10.3390/ijms22020727 33450900 PMC7828336

[45] Chen W. On nano-solutions to overcome cancer hypoxia and resistance. Nano TransMed. (2023) 2:e9130020. doi: 10.26599/NTM.2023.9130020

[46] Savage T Pandey S Guha C. Postablation modulation after single high-dose radiation therapy improves tumor control via enhanced immunomodulation. Clin Cancer Res: An Off J Am Assoc Cancer Res. (2020) 26:910–21. doi: 10.1158/1078-0432.CCR-18-3518 31757878

[47] Sun X-S Liu S-L Luo M-J Li X-Y Chen Q-Y Guo S-S. The association between the development of radiation therapy, image technology, and chemotherapy, and the survival of patients with nasopharyngeal carcinoma: A cohort study from 1990 to 2012. Int J Radiat Oncol Biol Physics. (2019) 105:581–90. doi: 10.1016/j.ijrobp.2019.06.2549 31319091

[48] Wang H Ouyang W Liu H. Tumor microenvironment responsive nanozymes for multimodal imaging of tumors. Nano TransMed. (2024) 3:100032. doi: 10.1016/j.ntm.2024.100032

[49] Dudek AM Martin S Garg AD Agostinis P. Immature, semi-mature, and fully mature dendritic cells: toward a DC-cancer cells interface that augments anticancer immunity. Front Immunol. (2013) 4:438. doi: 10.3389/fimmu.2013.00438 24376443 PMC3858649

[50] Guan G He Y Mei L. Atomic force microscopy: A nanobiotechnology for cellular research. Nano TransMed. (2022) 1:9130004. doi: 10.26599/NTM.2022.9130004

[51] Fu C Jiang A. Dendritic cells and CD8 T cell immunity in tumor microenvironment. Front Immunol. (2018) 9:3059. doi: 10.3389/fimmu.2018.03059 30619378 PMC6306491

[52] Gottfried E Kunz-Schughart LA Ebner S Mueller-Klieser W Hoves S Andreesen R. Tumor-derived lactic acid modulates dendritic cell activation and antigen expression. Blood. (2006) 107:2013–21. doi: 10.1182/blood-2005-05-1795 16278308

[53] Rajput IR Hussain A Li YL Zhang X Xu X Long MY. Saccharomyces boulardii and Bacillus subtilis B10 modulate TLRs mediated signaling to induce immunity by chicken BMDCs. J Cell Biochem. (2014) 115:189–98. doi: 10.1002/jcb.v115.1 24038094

[54] MaChado FC Girola N Maia VSC Bergami-Santos PC Morais AS Azevedo RA. Immunomodulatory protective effects of rb9 cyclic-peptide in a metastatic melanoma setting and the involvement of dendritic cells. Front Immunol. (2019) 10:3122. doi: 10.3389/fimmu.2019.03122 32010152 PMC6974543

[55] Yu N Wang S Song X Gao L Li W Yu H. Low-dose radiation promotes dendritic cell migration and IL-12 production via the ATM/NF-kappaB pathway. Radiat Res. (2018) 189:409–17. doi: 10.1667/RR14840.1 29420126

[56] Punnanitinont A Kannisto ED Matsuzaki J Odunsi K Yendamuri S Singh AK. Sublethal radiation affects antigen processing and presentation genes to enhance immunogenicity of cancer cells. Int J Mol Sci. (2020) 21(7):2573. doi: 10.3390/ijms21072573 32272797 PMC7178186

